# Comment on: ‘The proximal centriole age in spermatozoa is a potential reason for its different fate in the zygote after fertilization’ Uzbekov and Avidor-Reiss 2024

**DOI:** 10.1098/rsob.230458

**Published:** 2024-03-06

**Authors:** Rustem Uzbekov, Tomer Avidor-Reiss

**Affiliations:** ^1^ Laboratory of Cell Biology and Electron Microscopy, Faculty of Medicine, University of Tours, 37032 Tours, France; ^2^ Faculty of Bioengineering and Bioinformatics, Moscow State University, 119992 Moscow, Russia; ^3^ Department of Biological Sciences, University of Toledo, Toledo, OH 43606, USA; ^4^ Department of Urology, College of Medicine and Life Sciences, University of Toledo, Toledo, Ohio, USA

The article by Nils Kalbfuss & Pierre Gönczy [1] is devoted to one of the least explored issues of centrosome function in the cell—the mechanisms of centriole elimination. This commentary discusses previously described examples of such elimination and their significance for individual cells and animals as a whole in the context of reproduction. We propose to highlight one additional case in which centriole elimination may occur. In 2023, we described the process of centrosome formation in early mammals' development, taking zygotes and early cows’ embryos as the study object [2]. As our article shows, the sperm-derived proximal centriole was sometimes retained in the zygote but failed to duplicate a new canonical centriole.

In some zygotes, two polar corpuscles were located at each pole of the mitotic spindle, and the proximal centriole of the sperm was no longer detected. In some other zygotes, one of the poles had the proximal centriole. Similarly, in some other studies, centrioles were sometimes (but not always!) found at the poles of the first division [3,4]. How is the proximal centriole eliminated or whether it becomes a polar corpuscle, remains a mystery.

What could be the reason for the irregular presence of canonical centrioles at the poles of the zygote spindle? The article by Kalbfuss & Gönczy [1] allowed us to propose a hypothetical explanation for this phenomenon. In cow oocytes, centrioles are completely absent, as in all previously studied mammals [5,6]; they are eliminated during the process of oogenesis; the mechanism of this elimination is not fully understood.

Spermatids have two centrioles—proximal and distal. The proximal centriole is a more mature mother centriole, and the distal centriole is a daughter centriole, which was formed during the last cell cycle [7] (figure 1). A similar observation was made in fish [8].

The distal centriole gives rise to the flagellum and is transformed in the mature sperm into a funnel-shaped structure at the base of the flagellum with walls containing doublets of microtubules rather than triplets. The proximal centriole gives rise to spermatids' very specific structure—centriolar adjunct [9], which are similar in morphology to primary cilia in somatic cells. In mature sperm, the centriolar adjunct normally disappears, and the proximal centriole during fertilization is introduced in the zygote.

**Figure 1.**
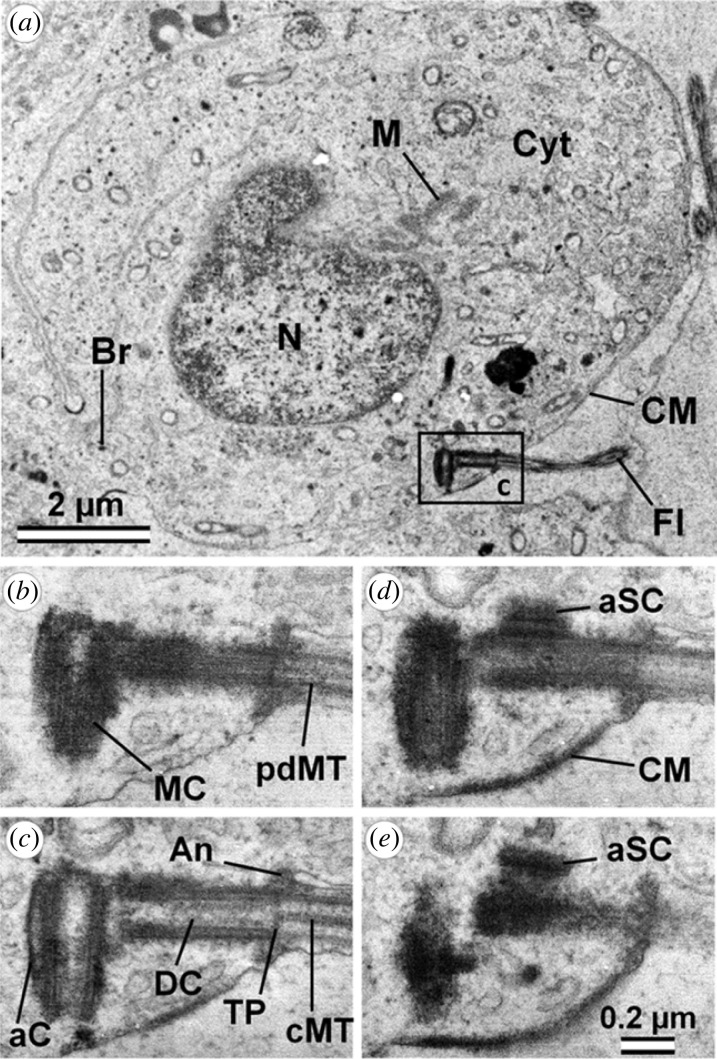
Ultrastructure of porcine early spermatid. (*a*) General view. (*b–e*) Four serial sections of centrosome region (indicated by frame in a). aC, anlage of capitulum; An, annulus; aSC, anlage of segmented columns; Br, cytoplasmic bridge between two adjacent spermatids; CM, cell membrane; cMT, central MT of axoneme; Cyt, cytoplasm; DC, daughter centriole; Fl, flagellum; M, mitochondria; MC, mother centriole; pdMT, peripheral doublets of MT of axoneme; TP, terminal plate between internal volumes of centriole and flagellum. Scale bars: (*a*) 2 µm; (*b*–*e*) 0.2 µm. Anlage of the capitulum later articulates with the implantation fossa of the spermatozoa nucleus; anlage of segmented (cross-striated) columns later forms the connecting piece of spermatozoa. From [7].

This situation, when both centrioles form structures containing axoneme, is unusual since, in most types of animal cells, only the mother centriole forms a single primary cilium [10,11].

Unlike the daughter centriole, which arose in the previous cell cycle, the age of the mother centriole can be very different. The mother centriole could have formed two, ten or even 50 cell cycles earlier! And during its life, this mother centriole could have accumulated different amounts of structural damage that can act as elimination factors in the zygote, as discussed in the article by Kalbfuss & Gönczy [1] (figure 2).

**Figure 2.**
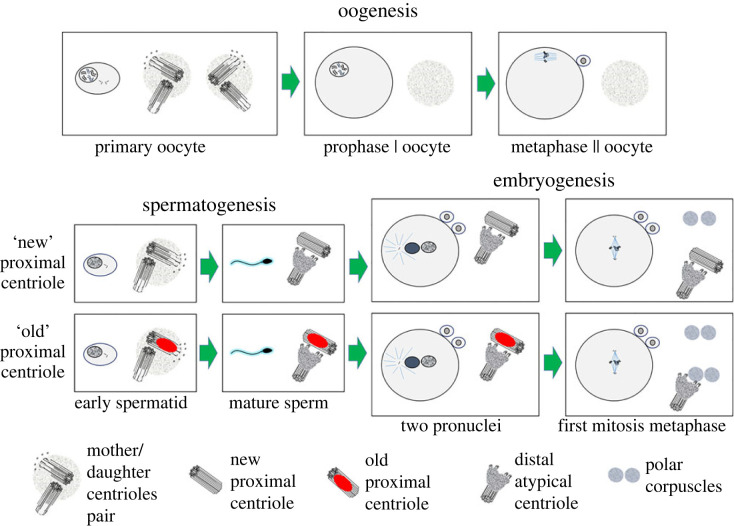
Hypotheses on the fate of centrioles in oogenesis, spermatogenesis and embryogenesis of mammals. In oogenesis, a pair of centrioles are present in primary oocytes but disappear completely during oocyte growth and maturation. During spermatogenesis, spermatids have a pair of centrioles—distal and proximal. In mature sperm, the distal centriole becomes atypical, and with the proximal centriole penetrates the oocyte during fertilization. Depending on the ‘age’ and the accumulation of elimination markers, the fate of the proximal centriole in the zygote and early embryo may be different. Old proximal centrioles are destroyed under the action of the elimination system preserved and replaced with a polar corpuscle in the zygote from the oocyte; new proximal centrioles that do not carry elimination markers can be preserved. In any case, regardless of the preservation or elimination of the proximal sperm centriole, the zygote genome initially produces atypical centrioles, which at the first stage have the form of polar corpuscles, which during embryogenesis are gradually transformed into centrioles of a canonical structure. The left side of each figure shows a general view of an oocyte, spermatid, sperm or embryo; the right side shows the region of the centrosome or spindle pole.

Sperm differ by the sex chromosome they carry, but they may also vary by the age of their proximal centriole! This fact may directly impact the proximal centriole fate after the sperm has been introduced into the zygote. If the proximal centriole has accumulated elimination factors (as described by Kalbfuss & Gönczy [1]) over its long life, then the mechanisms in the cytoplasm of the zygote can eliminate it.

Since centriole elimination occurs during oogenesis, the components of this elimination system are likely to remain present in the zygote cytoplasm. In our opinion, more important for the embryo's development is not the proximal centriole itself but the surrounding material of segmented columns, which form the sperm aster of microtubules [12].

For the passage of the first divisions in embryo development, the loss of the centriole is not critical for basic cell functions since, as our studies have shown, in parthenogenetically developing cow embryos, centrioles appear de novo already at the 4-blastomere stage [2]. Consequently, the genome has a program for building centrioles that are not associated with the material introduced by the sperm. However, parthenogenetically developing cow embryos fail to progress, suggesting they are abnormal [13].

In spermatids and spermatozoa-bearing younger proximal centrioles, elimination factors may not be sufficient to activate the process of centriole destruction.

Sperm is continually produced in a male individual for decades, and older men's sperm centrioles have reduced quality [14]. After all, unlike the DNA in chromosomes, cells do not appear to have centriole repair mechanisms, raising the question: are the spermatogonia stem cells that give rise to them periodically removed as damage and centriole elimination factors accumulate in them? The process of DNA repair after damage is described in detail [15]. As for centrioles, the process of centrioles restoration after damage has yet to be described. For example, centrioles partially destroyed in anaphase of mitosis by ultraviolet microirradiation are not restored, centriole duplication is blocked [16], and such cells exit from the cell cycle [17].

We propose that two types of actors are necessary for centriole elimination:

(i) A system for carrying out centriole elimination inherited from the oocyte.
(ii) Sperm centriole elimination factors that mark the centriole for elimination.

The nature of the centriole elimination is unclear. In fly oogenesis, PLK1 was identified as a regulator of centriole elimination [18], but this mechanism was not conserved in worms [19]. The nature of sperm centriole elimination factors is unknown, their presence can be tested using immunocytochemical analysis when identified, and specific antibodies against them are obtained.

## Data Availability

This article uses one figure from my previous publication. I have the right to use this image for my publications, provided that there is a link to it: https://www.researchgate.net/publication/327646211_Chapter_5_A_QUESTION_OF_FLAGELLA_ORIGIN_FOR_SPERMATIDS_-_MOTHER_OR_DAUGHTER_CENTRIOLE
